# The effect of T. aestivum chromosomes 1A and 1D on fertility of alloplasmic recombinant (H. vulgare)-T. aestivum lines depending on cytonuclear compatibility

**DOI:** 10.18699/vjgb-24-68

**Published:** 2024-10

**Authors:** Л.А. Першина, Н.В. Трубачеева, В.К. Шумный

**Affiliations:** Федеральный исследовательский центр Институт цитологии и генетики Сибирского отделения Российской академии наук, Новосибирск, Россия Курчатовский геномный центр ИЦиГ СО РАН, Новосибирск, Россия; Федеральный исследовательский центр Институт цитологии и генетики Сибирского отделения Российской академии наук, Новосибирск, Россия Курчатовский геномный центр ИЦиГ СО РАН, Новосибирск, Россия; Федеральный исследовательский центр Институт цитологии и генетики Сибирского отделения Российской академии наук, Новосибирск, Россия

**Keywords:** allolines (H. vulgare)-T. aestivum, chromosomes 1A and 1D, mtDNA, violation of cytonuclear compatibility, cytonuclear coadaptation, Rf genes, аллолинии (H. vulgare)-T. aestivum, хромосомы 1A и 1D, мтДНК, нарушение цитоядерной совместимости, цитоядерная коадаптация, гены Rf

## Abstract

The effect of T. aestivum L. chromosomes 1A and 1D on fertility of recombinant bread wheat allolines of the same origin carrying the cytoplasm of barley H. vulgare L. and different levels of cytonuclear compatibility was studied. Alloline L-56 included mainly fully sterile (FS) and partially sterile (PS) plants, alloline L-57 included partially fertile (PF) plants and line L-58 included fertile (F) ones. Analysis of morphobiological traits and pollen painting indicated complete or partial male sterility in plants of allolines L-56 and L-57. To differentiate genotypes with cytonuclear coadaptation and genotypes with cytonuclear incompatibility, PCR analysis of the 18S/5S mitochondrial (mt) repeat was performed. Heteroplasmy (simultaneous presence of barley and wheat mtDNA copies) was found in FS, PS, PF and some F plants, which was associated with a violation of cytonuclear compatibility. Wheat-type homoplasmy (hm) was detected in the majority of the fertile plants, which was associated with cytonuclear coadaptation. The allolines used as maternal genotypes were crossed with wheat-rye substitution lines 1R(1A) and 1R(1D). In F1, all plants of PF×1R(1A) and PF×1R(1D) combinations were fertile, and in F2, a segregation close to 3 (fertile) : 1 (sterile) was observed. These results showed for the first time that chromosomes 1A and 1D carry one dominant Rf gene, which controls the restoration of male fertility of bread wheat carrying the cytoplasm of H. vulgare. All plants of F1 combinations FS×1R(1A), FS×1R(1D), PS×1R(1A), PS×1R(1D) were sterile, which indicates that a single dose of genes localized on wheat chromosomes 1A or 1D is not enough to restore male fertility in FS and PS plants. All plants of hybrid combinations F(hm)×1R(1A) and F(hm)×1R(1D) in both F1 and F2 were fertile, that is, fertility of allolines with cytonuclear coadaptation does not depend on wheat chromosomes 1A and 1D.

## Abstract (ru)

Изучено влияние хромосом 1A и 1D T. aestivum L. на фертильность рекомбинантных аллолиний мягкой пшеницы одного происхождения, имеющих цитоплазму ячменя H. vulgare L. и разный уровень цитоядерной совместимости. Аллолиния Л-56 включает преимущественно полностью стерильные (ПС) и частично стерильные (ЧС) растения; аллолиния Л-57 – частично фертильные (ЧФ) растения, а линия Л-58 – фертильные (Ф) растения. Результаты анализа морфобиологических признаков и окраски пыльцы указывают на проявление полной или частичной мужской стерильности у растений аллолиний Л-56 и Л-57. Для разделения генотипов с цитоядерной коадаптацией и генотипов, у которых цитоядерная совместимость нарушена, выполнен ПЦР-анализ 18S/5S митохондриального (мт) повтора. Показано, что ПС, ЧС, ЧФ и часть Ф растений характеризуются гетероплазмией (наличием копий мтДНК ячменя и пшеницы), что ассоциировано с нарушением цитоядерной совместимости. У основной части фертильных растений выявлена гомоплазмия (гм) пшеничного типа, что ассоциировано с цитоядерной коадаптацией. Растения аллолиний, использованные в качестве материнских генотипов, были скрещены с пшенично-ржаными замещенными линиями 1R(1A) и 1R(1D). В F1 все растения комбинаций ЧФ×1R(1A) и ЧФ×1R(1D) были фертильными, а в F2 наблюдали расщепление, близкое к 3 (фертильные) : 1 (стерильные). Эти результаты впервые показали, что в хромосомах 1A и 1D локализовано по одному доминантному гену Rf, контролирующему восстановление мужской фертильности мягкой пшеницы, несущей цитоплазму H. vulgare. Все растения F1 комбинаций ПС×1R(1A), ПС×1R(1D), ЧС×1R(1A), ЧС×1R(1D) стерильные, что указывает на то, что одной дозы генов, локализованных в хромосомах пшеницы 1A или 1D, недостаточно для восстановления мужской фертильности у ПС и ЧС растений. Все растения гибридных комбинаций Фгм×1R(1A) и Фгм×1R(1D) и в F1 и в F2 были фертильными, т. е. у аллолиний с цитоядерной коадаптацией нет зависимости проявления фертильности от влияния хромосом пшеницы 1A и 1D.

## Introduction

Alloplasmic lines (allolines) are resulted from repeated crosses of wide F1 hybrids with a pollen parent. These lines combine the cytoplasm from the maternal species with the nuclear genome from the paternal species (Tsunewaki, 1996). The replacement of cytoplasm affects nuclear-mitochondrial and nuclear-chloroplast interactions (Yang et al., 2008; Crosatti et al., 2013; Soltani et al., 2016) leading to changes in plant development (Badaeva et al., 2006), resistance to stress factors (Buloychik et al., 2002; Talukder et al., 2015; Takenaka et al., 2019), morphological and agronomic traits (Liu C.G. et al., 2002; Atienza et al., 2008; Tao et al., 2011; Klimushina et al., 2013). The most relevant manifestation of cytonuclear conflict is cytoplasmic male sterility (CMS) (Tsunewaki, 1996), which is associated with aberrant mitochondrial genes that negatively affect the development of flower and pollen organs (Yang et al., 2008).

In a number of economically important crops, CMS lines in combination with maintainer and restorer lines carrying male fertility restoration genes (Rf – restorer-of-fertility) have been used in hybrid breeding (Islam et al., 2014; Bohra et al., 2016; Gupta et al., 2019). The sources of CMS and restorer genes are a critical tool in this technology. In addition, cytoplasmic substitution results in an increase of cytoplasmic diversity, as has been shown for crops such as rice (Liu Y. et al., 2016), sugar cane (Rafee et al., 2010), and bread wheat (Liu C.G. et al., 2002; Klimushina et al., 2013; Pershina et al., 2018).

In this regard, studying the process of allolines development and the genetic control of fertility restoration is an important task both for identifying new CMS-Rf systems for hybrid breeding and for obtaining new genotypes for conventional breeding programs. In bread wheat, male fertility restoration of genotypes carrying the cytoplasm of T. timopheevii (Sinha et al., 2013), H. chilense (Martin et al., 2010), Aegilops species (Tsunewaki, 2015; Hohn, Lukaszewski, 2016) and cultivated barley H. vulgare (Pershina et al., 2012; Trubacheeva et al., 2021) has been studied. Most Rf genes in bread wheat were located in clusters on chromosomes of the homeologous groups 1, 2 and 6, and the largest number was located on chromosome 1 (Gupta et al., 2019).

In a previous study, we established for the first time that the dominant gene controlling the male fertility restoration of wheat carrying H. vulgare cytoplasm was located on the short arm of wheat chromosome 1B (Trubacheeva et al., 2021). In this work, the role of homeologous group 1 for fertility restoration of bread wheat allolines carrying H. vulgare cytoplasm continues to be studied. The aim of the work was to study the effect of T. aestivum chromosomes 1A and 1D on the male fertility of recombinant wheat allolines carrying cultivated barley cytoplasm depending on the level of their fertility and cytonuclear compatibility. This approach allowed us to identify allolines (H. vulgare)-T. aestivum as models for studying the localization of the Rf genes on chromosomes 1A and 1D.

## Materials and methods

**Fig. 1.**
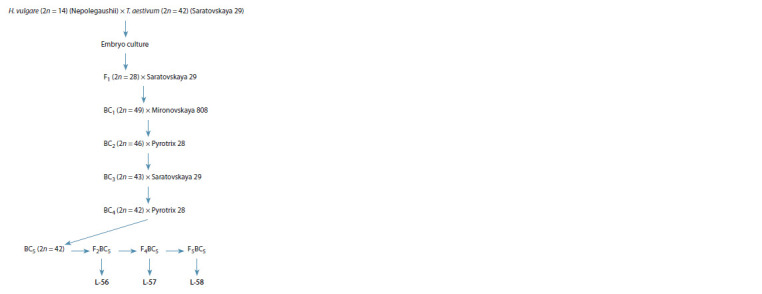
Production of the alloplasmic recombinant lines (H. vulgare)- T. aestivum L-56, L-57, L-58.

Plant material. Three recombinant allolines (H. vulgare)- T. aestivum derived from individual plants of backcross (BC) generations of a barley-wheat hybrid H. vulgare (Nepolegaushii) × T. aestivum (Saratovskaya 29), sequentially pollinated with wheat varieties Saratovskaya 29, Mironovskaya 808, Pyrotrix 28, Saratovskaya 29, Pyrotrix 28, were studied (Fig. 1). In previous studies, Saratovskaya 29 was found to be a sterility fixer in backcrossed progenies of barley-wheat hybrids (Pershina et al., 2012), while Mironovskaya 808 and Pyrotrix 28 were identified as male fertility restorers for wheat alloplasmic lines carrying cultivated barley cytoplasm (Pershina et al., 1998, 2012). BC1–BC4 generations and the barley-wheat hybrid were male-sterile, but female-fertile, and in ВС5, some 42-chromosomal plants with partially restored male fertility were isolated. Self-pollinated generations F2ВС5–F5ВС5 were obtained from these plants, which became the sources of the studied allolines. Alloline L-56 was isolated from F2BC5, and allolines L-57 and L-58 were isolated from F4BC5 and F5BC5, respectively. Beginning from F3BC5, plants with the highest level of productivity were used to obtain each subsequent self-pollinated generation

Methods for studying morphobiological characteristics of alloplasmic recombinant lines. Plants of the lines used were characterized by fertility level: FS – fully sterile (no seeds); PS – partially sterile (1–9 seeds); PF – partially fertile (10–19 seeds); F – fertile (more than 19 seeds per main spike). At least 20 plants of each line grown in a hydroponic greenhouse were evaluated.

Pollen fertility as the main criterion for assessing male fertility/sterility was analyzed in plants with different fertility levels. For this purpose, crushed preparations in Lugol’s solution (1 % iodine solution in an aqueous solution of potassium iodide) were prepared on a slide from anthers isolated during flowering from three different flowers of the same spike. Plant height, the number of spikes, main spike length, the number of spikelets per main spike, grain number per main spike and per plant, and 1,000-grain weight were determined for the plants of each alloline. The differences between the average values of the studied traits in alloline L-56 compared with the L-57 line and in alloline L-57 compared with alloline L-58 were statistically evaluated by Student’s t-test. Data were analyzed using Statistica v.7.0.61.0.

PCR analysis of the 18S/5S mitochondrial (mt) repeat. Specific primers for the 18S/5S repeat were designed based on the mitochondrial genome sequences published earlier (Coulthart et al., 1993). The PCR products were electrophoresed in a 1.5 % agarose gel with 1× TAE buffer and visualized with ethidium bromide. Gel images were captured using the gel documentation system Gel Doc XR+ (“Bio- Rad”, USA). Total DNA was isolated from green leaves cut before earing according to a previously published protocol (Current Protocols…, 1987). From one to eight samples from individual genotypes were analyzed. In this part of the work, the control was the barley variety Nepolegaushii as a source of cytoplasm for allolines and the bread wheat variety Pyrotrix 28 as a source of wheat cytoplasm (one of the recurrent genotypes).

Evaluation of the fertility of hybrids between alloplasmic lines and wheat-rye substitution lines 1A(1R) and 1D(1R) in F1 and F2. To assess the effect of wheat chromosomes 1A and 1D on the fertility of allolines depending on the level of their cytonuclear compatibility, plants of these lines (as maternal genotypes) were crossed with wheat-rye substitution lines 1A(1R) and 1D(1R) to replace in F1 one 1A or 1D chromosome of allolines with rye chromosome 1R. The 1A(1R) and 1D(1R) lines used in the work were obtained as a result of substituting wheat Saratovskaya 29 chromosomes with rye chromosome 1R of variety Onokhoyskaya (Shchapova, Kravtzova, 1982). In hybridization, FS and PS plants of alloline L-56, PF plants of alloline L-57 and some F plants of alloline L-58 were used. The spikes of mother plants, as well as F1 and F2 plants grown in a hydroponic greenhouse, were bagged before flowering. In individual plants of F1 and F2, the seed set in the main spike was assessed. Based on the seed set in the F2 generation, the individual plants were classified into fertile and sterile groups according to the recommendations of P. Sinha et al. (Sinha et al., 2013): completely sterile plants and plants that set no more than four grains in the main spike were classified as sterile, while those that set five or more grains in the main spike were classified as fertile. Pearson’s chi-squared test (α = 0.05) was used for the deviation of the observed data from the theoretically expected segregation into fertile and sterile plants in F2.

## Results

Characteristics of the recombinant allolines

Alloline L-56 consisted of partially sterile (60 %) and completely sterile plants (35 %); the frequency of partially fertile plants was 5 % (Table 1).

**Table 1.**
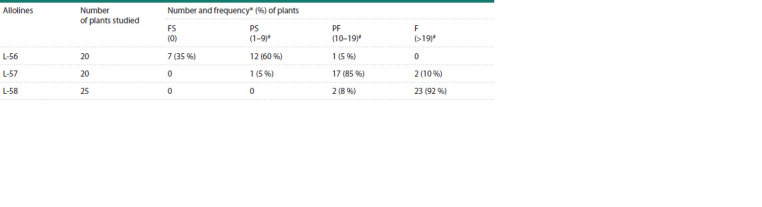
Fertility level of recombinant allolines (H. vulgare)-T. aestivum L-56, L-57, L-58 Notе. FS – full sterility; PS – partial sterility; PF – partial fertility; F – fertility. # – grain number per main spike.

**Fig. 2.**
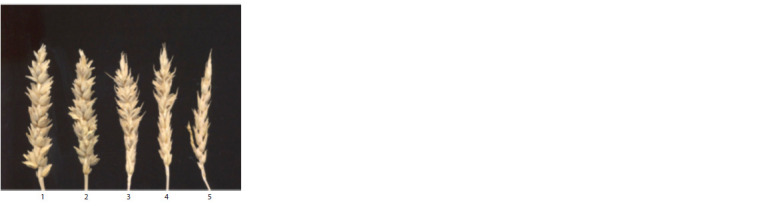
Plant spikes: 1, 2 – fertile; 3 – partially fertile; 4 – partially sterile; 5 – fully sterile.

The majority of plants in alloline L-57 were partially fertile (85 %), the rest were partially sterile (5 %) and fertile (10 %). Alloline L-58 consisted of fertile (92 %) and partially fertile plants (8 %). Figure 2 shows plant spikes with different fertility levels.

**Fig. 3.**
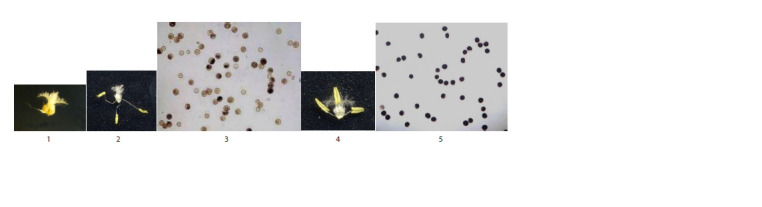
Stigma (1) of a fully sterile plant; stigma and anthers (2), pollen grains (3) of a partially fertile plant; stigma and anthers (4) and pollen grains (5) of a fertile plant.

In fully sterile plants, stigmas were normally developed, but anthers were absent. In partially sterile and partially fertile plants, anthers were not fully developed compared to fertile plants, and not all pollen grains were stained (Fig. 3).

The comparison of the average values of the studied traits in L-56, represented mainly by sterile and partially sterile plants, compared with L-57, consisting mainly of partially fertile plants, showed that L-56 exceeded L-57 only in terms of the number of spikes per plant. The value of other traits (plant height, length of the main spike, number of spikelets per main spike, grain number per main spike and per plant) in L-56 is significantly lower than in L-57 (Table 2).

**Table 2.**
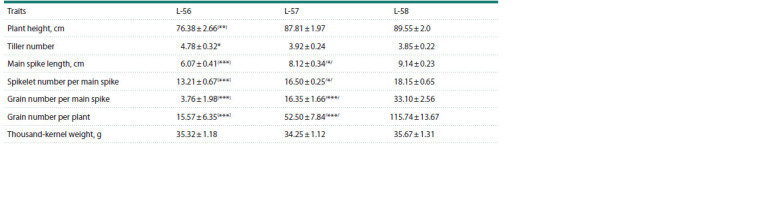
Agronomic characteristics of recombinant (H. vulgare)-T. aestivum lines Notе. The difference compared to L-57 is significantly greater at * p < 0.05; significantly less at (**) p < 0.01 and (***) p < 0.001; compared with L-58, significantly less at /*/ p < 0.05 and /***/ p < 0.001.

In alloline L-57, the values of main spike length, number of spikelets per main spike, grain number per main spike and per plant were significantly lower compared to alloline L-58, represented mainly by fertile plants. Thousand-kernel weight did not differ between the studied allolines.

PCR analysis of 18S/5S mtDNA in recombinant allolines

**Fig. 4.**
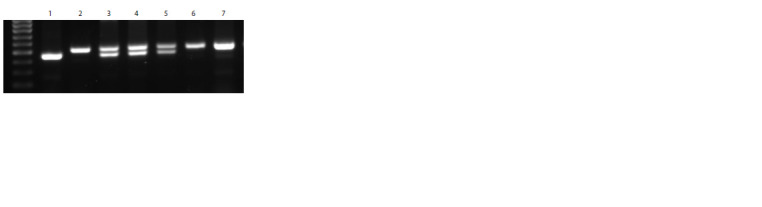
Agarose gel electrophoresis of PCR products using the 18S/5S mt repeat marker 1 – barley H. vulgare variety Nepolegaushii; 2 – wheat T. aestivum variety Pyrotrix 28; 3 – completely sterile L-56 plant; 4 – partially sterile L-56 plant; 5 – partially fertile L-57 plant; 6, 7 – fertile L-58 plants.

Heteroplasmy (simultaneous presence of barley and wheat mtDNA copies) was found in all studied plants of alloline L-56, including fully sterile, partially sterile plants and one partially fertile plant (Fig. 4; Table 3). Heteroplasmy was also detected in six partially fertile and two fertile plants of alloline L-57. In alloline L-58, two partially fertile and two fertile plants were found to have heteroplasmy, and six fertile plants had wheat-type homoplasmy. These results were used to divide alloplasmic genotypes into groups with different levels of cytonuclear incompatibility according to the data (Aksyonova et al., 2005; Trubacheeva et al., 2021) (Table 3). In plants with heteroplasmy, cytonuclear compatibility was disrupted, while in plants with homoplasmy it was not.

**Table 3.**
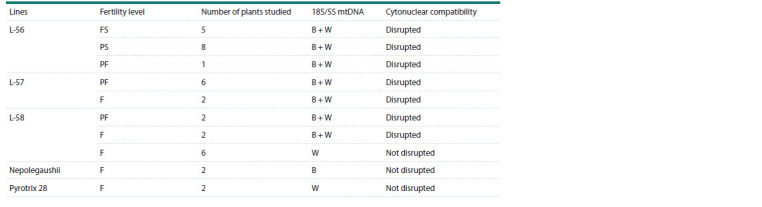
Results of the study of the 18S/5S mtDNA repeat in recombinant allolines (H. vulgare)-T. aestivum Notе. B – barley; W – wheat; Nepolegaushii is a variety of barley; Pyrotrix 28 is a variety of bread wheat.

Analysis of hybrids between recombinant allolines and wheat-rye substitution lines 1R(1A) and 1R(1D)

Individual fully sterile (FS) and partially sterile (PS) plants of alloline L-56 were pollinated with pollen of wheat-rye

substitution lines 1R(1A) and 1R(1D). Seeds were set in all combinations of crossing due to female fertility of FS and PS plants. F1 plants were grown from the set seeds: 18 plants of combination L-56(FS) × 1R(1A), 20 plants of combination L-56(FS) × 1R(1D), 15 plants of combination L-56(PS) × 1R(1A) and 17 plants of combination L- 56(PS) × 1R(1D). All F1 plants of these hybrid combinations did not set seeds from self-pollination (Table 4).

**Table 4.**
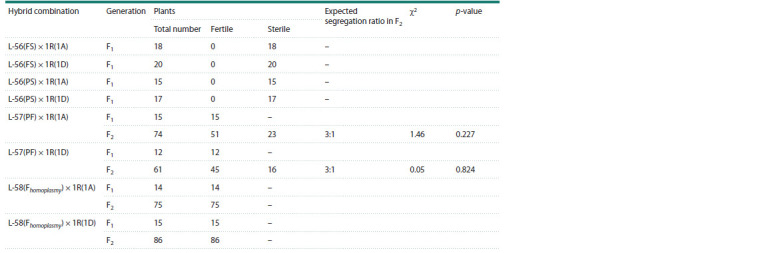
Seed setting in F1 hybrids and segregation for seed setting in F2 hybrids derived from the crossing of allolines (H. vulgare)-T. aestivum with wheat-rye substitution lines 1R(1A) and 1R(1D) of variety Saratovskaya 29

The complete sterility of F1 hybrids heterozygous for wheat chromosomes 1A and 1D indicates that the fertility of partially sterile plants depends on chromosomes 1A and 1D. However, a single dose of the gene localized on each of these chromosomes is not sufficient to restore the male fertility of these plants.

Partially fertile (PF) plants of alloline L-57 were included in hybridization with wheat-rye substitution lines. Fifteen plants were grown from seeds of the hybrid combination L-57(PF) × 1R(1A), and twelve F1 plants were grown from the combination L-57(PF) × 1R(1D). All F1 plants were fertile, which indicates that fertility restoration in these allolines is a dominant trait. The analysis of the seed set in the main spike of 74 F2 plants of the hybrid combination L- 57(PF) × 1R(1A) revealed 51 plants that were classified as fertile and 23 plants – as sterile. The observed ratio when segregated into fertile and sterile plants in F2 fitted well with the theoretically expected segregation ratio of 3 (fertile) : 1 (sterile) with an χ2 value of 1.46, which is lower than the statistical value of χ2 05 = 3.84.

A similar result was obtained for the hybrid combination L-57(PF) × 1R(1D). Out of the 61 F2 plants studied in this combination, 45 were classified as fertile and 16 were sterile, resulting in a value of χ2 = 0.05 (Table 4). These results indicated that the fertility of L-57 was dependent on wheat chromosomes 1A and 1D. The ratio of fertile and sterile plants in F2 of the combinations L-57(PF) × 1R(1A) and L-57(PF) × 1R(1D) showed that male fertility restoration in partially fertile plants of L-57 was controlled by a single dominant gene. One of these genes is localized on chromosome 1A, and the other, on chromosome 1D.

A different result was obtained when crossing fully fertile plants of L-58, in which wheat-type homoplasmy was detected, with wheat-rye substitution lines. All F1 and F2 plants of the combinations L-58(F) × 1R(1A) and L-58(F) × 1R(1D) were fertile (Table 4). This means that the fertility of alloline L-58 included in crosses with wheat-rye substitution lines does not depend on bread wheat chromosomes 1A and 1D.

## Discussion

There is a strong intergenomic incompatibility between cultivated barley H. vulgare and bread wheat T. aestivum, which prevents both crossing between them and fertility restoration of hybrids. However, due to the use of methods to overcome incompatibility and the selection of parental genotypes, it was possible to obtain viable barley-wheat F1 hybrids with female fertility (Pershina et al., 1998). This made it possible to include hybrids in backcrosses with different varieties of bread wheat leading to the elimination of barley chromosomes, the creation of a recombinant wheat nuclear genome and the replacement of a wheat cytoplasm with the cytoplasm of barley in alloplasmic genotypes (Aksyonova et al., 2005; Pershina et al., 2012).

The recombinant allolines (H. vulgare)-T. aestivum L-56, L-57 or L-58 had the same origin, but differed in morphobiological characteristics and fertility level. The recombinant nuclear genome of these lines was obtained using the varieties of bread wheat Saratovskaya 29, Mironovskaya 808, and Pyrotrix 28. The expression of morphobiological traits in the L-56 line, compared to the L-57 line, represented by partially fertile plants, was suppressed. The L-56 alloline segregated into fully sterile plants and plants with a low fertility level. Apparently, the genome of Saratovskaya 29 prevailed in the nuclear genome of the L-56 line. This variety was a fixer of sterility of bread wheat carrying the cytoplasm of cultivated barley (Pershina et al., 2012).

The absence of anthers in fully sterile plants and incomplete staining of pollen grains in partially fertile plants was caused by CMS, which resulted from disruption of nuclear-mitochondrial interactions (Yang et al., 2008). PCR analysis of the 18S/5S mt repeat in the L-56 and L-57 allolines revealed heteroplasmy, that is, the coexistence of two mtDNA variants, the barley and the wheat type. Heteroplasmy of mtDNA in barley-wheat hybrids and allolines derived from them is a consequence of biparental transmission of mtDNA beginning from F1 (Aksyonova et al., 2005). This phenomenon has been described for hybrids (Ae. crassa × wheat Chinese Spring) (Kawaura et al., 2011) and allolines (Ae. longissima)-T. turgidum (Noyszewski et al., 2014). Inheritance of cytoplasmic genomes from both parents, compared with strictly maternal one, results in a greater diversity of mt- and cpDNA variants in hybrids. It has been suggested that biparental inheritance of chloroplasts in angiosperms leads to rescue species with defective plastids (Zhang, Sodmergen, 2010). This mechanism can also reduce the negative impact of cytonuclear incompatibility on the development of F1 hybrids (Barnard-Kubow et al., 2016). It can be assumed that in the L-56 and L-57 allolines, the presence of wheat copies of mtDNA, along with barley copies, was also a manifestation of neutralization of the cytonuclear conflict between barley cytoplasm and wheat nuclear genome, ensuring the development of viable allolines, albeit with reduced fertility.

When backcrossing hybrids with mtDNA heteroplasmy with the paternal species (wheat), variability was found not only in the nuclear genome, but also in the mitochondrial genome (Aksyonova et al., 2005; Trubacheeva et al., 2012, 2021). When the fertility of the allolines was restored, the number of mtDNA copies of the wheat (paternal) type increased and the original alloplasmic condition appeared to be lost (Aksyonova et al., 2005; Trubacheeva et al., 2021). The same process was observed in the production of wheat allolines carrying the cytoplasm of some Aegilops species (Tsukamoto et al., 2000; Hattori et al., 2002). The fully fertile L-58 alloline without CMS was isolated by selecting plants with maximum fertility in the F5BC5 generation of the barley-wheat hybrid (Fig. 1). It can be assumed that in L-58, the recombinant nuclear genome without barley chromosomes contains mainly the genomes of the wheat varieties Mironovskaya 808 and Pirotrix 28, which are restorers of fertility in bread wheat with the cytoplasm of cultivated barley (Pershina et al., 2012, 2018). During selection for fertility, as well as during backcrossing, the variability of mtDNA from heteroplasmy to wheat-type homoplasmy correlates with the variability of chloroplast DNA from barley-type homoplasmy to wheat-type homoplasmy (Aksyonova et al., 2005; Trubacheeva et al., 2021).

As follows from the data obtained both in this work and in previously published ones (Aksyonova et al., 2005; Trubacheeva et al., 2012, 2021), heteroplasmy and wheat-type homoplasmy detected in allolines can be used as markers for dividing allolines into groups with cytonuclear incompatibility and cytonuclear coadaptation, since it is not in all cases that the fertility level can be a reliable trait for such a division. For example, in both this and a previously published work (Trubacheeva et al., 2021), mtDNA heteroplasmy was found in some fertile plants, that is, there was a violation of cytonuclear compatibility.

Clear differences between allolines with cytonuclear incompatibility and cytonuclear coadaptation were found when studying the effect of chromosomes 1A and 1D on the fertility of these lines. In allolines L-56 and L-57 with cytonuclear incompatibility, male fertility depends on these wheat chromosomes, but in alloline L-58 without cytonuclear incompatibility, it does not. This can be explained by the fact that in allolines L-56 and L-57 with heteroplasmy, the Rf genes located on chromosomes 1A and 1D are necessary to neutralize the sterilizing effect of the cytoplasm. In line L-58 with cytonuclear coadaptation, wheat-type cytoplasm was formed, so the production of male-fertile plants did not depend on the presence of the Rf genes on these chromosomes.

We observed similar differences in our previous work (Trubacheeva et al., 2021): the short arm of chromosome 1B affected the fertility of the allolines with cytonuclear incompatibility, but did not affect the fertility of the allolines with cytonuclear compatibility.

## Conclusion

To perform this work, among the backcrossed progenies of the barley-wheat hybrid H. vulgare × T. aestivum, sequentially pollinated with different varieties of bread wheat, three allo-lines of bread wheat with the cytoplasm of cultivated barley were isolated. These allolines of the same origin but differed by fertility and cytonuclear compatibility were used as adequate models to determine the localization of genes controlling the restoration of fertility of bread wheat carrying the cytoplasm of cultivated barley.

Based on the results of segregation in F2 hybrids obtained from crossing alloline L-57 with wheat-rye substitution lines 1R(1A) and 1R(1D), it was concluded for the first time that chromosomes 1A and 1D carry one dominant Rf gene, which controls male fertility restoration of bread wheat with the cytoplasm of cultivated barley. However, a single dose of these genes is not enough to restore the fertility of partially sterile plants. The results of our work supplemented the information on the localization of the Rf genes in wheat chromosomes 1A, 1D (this work) and 1BS (Trubacheeva et al., 2021).

An important finding was that the fertility of the line with cytonuclear compatibility did not depend on the chromosomes in which the Rf genes were located. This explains the fact that the introgression of alien germplasm into the lines, including the replacement of the short arm of wheat chromosome 1B by the short arm of the rye chromosome 1R, does not violate cytonuclear compatibility, and allolines maintain fertility (Pershina et al., 2018, 2020). Moreover, based on introgression (H. vulgare)-T. aestivum allolines, DH lines were obtained and used as maternal genotypes to develop commercial high-yielding spring wheat varieties Sigma, Uralosibirskaya 2, Sigma 5 (Belan et al., 2021).

## Conflict of interest

The authors declare no conflict of interest.
